# Hypermethylation of the TGF-β target, *ABCA1* is associated with poor prognosis in ovarian cancer patients

**DOI:** 10.1186/s13148-014-0036-2

**Published:** 2015-01-14

**Authors:** Jian-Liang Chou, Rui-Lan Huang, Jacqueline Shay, Lin-Yu Chen, Sheng-Jie Lin, Pearlly S Yan, Wei-Ting Chao, Yi-Hui Lai, Yen-Ling Lai, Tai-Kuang Chao, Cheng-I Lee, Chien-Kuo Tai, Shu-Fen Wu, Kenneth P Nephew, Tim H-M Huang, Hung-Cheng Lai, Michael W Y Chan

**Affiliations:** Department of Life Science, National Chung Cheng University, 168 University Road, Min-Hsiung, Chia-Yi 621, Taiwan; Institute of Molecular Biology, National Chung Cheng University, Min-Hsiung, Chia-Yi, Taiwan; Division of Gastroenterology, Chang Gung Memorial Hospital, Chia-Yi, Taiwan; Division of Hematology, Department of Internal Medicine, Comprehensive Cancer Center, The Ohio State University, Columbus, OH USA; Department of Life Science, Tunghai University, Taichung, Taiwan; Medical Sciences, Department of Cellular and Integrative Physiology, Indiana University School of Medicine, Bloomington, IN USA; Department of Molecular Medicine, Institute of Biotechnology, University of Texas Health Science Center, San Antonio, TX USA; Department of Obstetrics and Gynecology, Shuang Ho Hospital, Taipei Medical University, No 291, Zhongzheng Rd., Zhonghe District, New Taipei City, 23561 Taiwan; Department of Pathology, Tri-Service General Hospital, National Defense Medical Center, Taipei, Taiwan; Department of Obstetrics and Gynecology, School of Medicine, College of Medicine, Taipei Medical University, Taipei, Taiwan; Department of Clinical Pharmacology, Xiangya Hospital; Institute of Clinical Pharmacology, Central South University and Hunan Key Laboratory of Pharmacogenetics, Changsha, China

**Keywords:** Ovarian cancer, Epigenetics, ABCA1

## Abstract

**Background:**

The dysregulation of transforming growth factor-β (TGF-β) signaling plays a crucial role in ovarian carcinogenesis and in maintaining cancer stem cell properties. Classified as a member of the ATP-binding cassette (ABC) family, *ABCA1* was previously identified by methylated DNA immunoprecipitation microarray (mDIP-Chip) to be methylated in ovarian cancer cell lines, A2780 and CP70. By microarray, it was also found to be upregulated in immortalized ovarian surface epithelial (IOSE) cells following TGF-β treatment. Thus, we hypothesized that *ABCA1* may be involved in ovarian cancer and its initiation.

**Results:**

We first compared the expression level of *ABCA1* in IOSE cells and a panel of ovarian cancer cell lines and found that *ABCA1* was expressed in HeyC2, SKOV3, MCP3, and MCP2 ovarian cancer cell lines but downregulated in A2780 and CP70 ovarian cancer cell lines. The reduced expression of *ABCA1* in A2780 and CP70 cells was associated with promoter hypermethylation, as demonstrated by bisulfite pyro-sequencing. We also found that knockdown of *ABCA1* increased the cholesterol level and promoted cell growth *in vitro* and *in vivo*. Further analysis of *ABCA1* methylation in 76 ovarian cancer patient samples demonstrated that patients with higher *ABCA1* methylation are associated with high stage (*P* = 0.0131) and grade (*P* = 0.0137). Kaplan-Meier analysis also found that patients with higher levels of methylation of *ABCA1* have shorter overall survival (*P* = 0.019). Furthermore, tissue microarray using 55 ovarian cancer patient samples revealed that patients with a lower level of ABCA1 expression are associated with shorter progress-free survival (*P* = 0.038).

**Conclusions:**

*ABCA1* may be a tumor suppressor and is hypermethylated in a subset of ovarian cancer patients. Hypermethylation of ABCA1 is associated with poor prognosis in these patients.

**Electronic supplementary material:**

The online version of this article (doi:10.1186/s13148-014-0036-2) contains supplementary material, which is available to authorized users.

## Background

Ovarian cancer is the most lethal tumor in women and the second most frequent gynecological malignancy [1]. As ovarian cancer has few symptoms early in its course, patients are generally diagnosed with advanced-stage tumors. Despite advances in chemotherapy, the poor prognosis for patients with late-stage ovarian cancer is reflected by the 5-year survival rate of less than 20% [2]. Current prognostic indicators using clinicopathological variables, including stage and grade, neither accurately predict clinical outcomes nor provide biological insight into the disease. Thus, a better understanding of the molecular changes in ovarian cancer would provide insight for better diagnosis and prognosis of this deadly disease.

The transforming growth factor-β (TGF-β) signaling pathway plays an important role in the regulation of ovarian growth [3]. During ovulation, the ovarian surface epithelial (OSE) cell covering the ovary undergoes rupture, followed by proliferation-mediated repair. This growth is inhibited by TGF-β, which plays a key role in preventing overproliferation of OSE cells [4]. In this regard, the dysregulation of TGF-β signaling may be an early step in the development of epithelial ovarian cancer. This is in agreement with the observation that resistance to TGF-β signaling is commonly seen in ovarian cancer, suggesting that reduced responsiveness to TGF-β is a key event in ovarian cancer [5].

Epigenetic alterations, including DNA methylation and histone modifications, play an important role in controlling gene expression [6,7]. Moreover, in cancer, aberrant epigenetic modifications are frequently found to be responsible for the silencing of tumor suppressor genes. Our previous studies using genome-wide techniques have identified several direct targets of TGF-β in immortalized ovarian surface epithelial (IOSE) cells [8]. These targets are silenced epigenetically in ovarian cancer and are associated with poor prognosis in ovarian cancer patients [9-11]. Restoration of the genes inhibited tumor growth, thus suggesting that they are tumor suppressors.

In order to identify additional genes that are hypermethylated in ovarian cancer, we performed methylated DNA immunoprecipitation microarray (mDIP-Chip) in IOSE cells and a panel of ovarian cancer cell lines. Our result showed that *ABCA1*, which is upregulated by TGF-β [8] and is a major regulator of cellular cholesterol [12], is hypermethylated in a subset of ovarian cancer cell lines. Further analysis demonstrated that *ABCA1* methylation was associated with poor prognosis in ovarian cancer patients. These results suggested that *ABCA1* may be a tumor suppressor and can serve as a prognostic indicator in ovarian cancer.

## Results

### *ABCA1* is epigenetically silenced in ovarian cancer cells

Our previous studies demonstrated that novel TGF-β targets, *FBXO32* and *RunX1T1*, are epigenetically silenced by promoter hypermethylation in ovarian cancer [10,11]. To identify additional genes regulated by TGF-β that exhibited promoter hypermethylation in ovarian cancer, we performed mDIP-Chip in IOSE cells and a panel of ovarian cancer cell lines [13]. One of the genes, *ABCA1*, which exhibited promoter methylation in a subset of ovarian cancer cell lines but not in IOSE cells, was selected for further analysis. This gene was also previously found to be upregulated in IOSE cells after TGF-β treatment [8].

**Figure 1:**
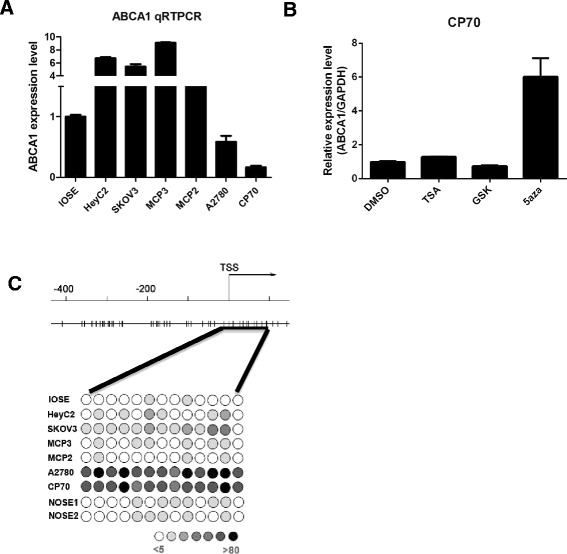
***ABCA1*** **expression and methylation level in IOSE cells and ovarian cancer cell lines. (A)** Total RNA was isolated from ovarian cells and converted into cDNA for amplification with specific primers for *ABCA1*. The relative level of expression after quantitative real-time RT-PCR was compared to IOSE cells (set as one fold). Each bar represents mean ± SD. **(B)** CP70 cells were treated with TSA (0.5 μM, 12 h), GSK343 (1 μM, 3 days), or 5aza (0.5 μM, 3 days). The expression level of ABCA1 was determined by RT-PCR. Treatment of 5aza, but not TSA or GSK, resulted in robust re-expression of *ABCA1* in CP70 cells. Each bar represents mean ± SD. **(C)** The methylation status of the *ABCA1* promoter and TSS region was analyzed by bisulfite pyro-sequencing from −90 to +190 (black line underneath). The upper panel shows the *ABCA1* promoter and TSS region and the corresponding CpG sites (vertical bar), and the lower panel illustrates DNA methylation at the interrogated CpG site (circle) in IOSE cells, two NOSE samples, and ovarian cancer cell lines with intensity of gray color indicating methylation level.

To confirm our microarray result, we first examined the mRNA expression level of *ABCA1* in various ovarian cancer cell lines (Figure 1A). The result showed that *ABCA1* was repressed in A2780 and CP70 cells. Although treatment of HDAC inhibitor (TSA) or EZH2 inhibitor (GSK343) alone did not result in any significant re-expression of *ABCA1*, treatment of demethylating agent, 5aza, alone resulted in a robust re-expression of *ABCA1* in CP70 ovarian cancers, suggesting that DNA methylation is responsible for the suppression of *ABCA1* in CP70 cells (Figure 1B). These results were confirmed by bisulfite pyro-sequencing such that CpG sites around the transcriptional start site (TSS) of *ABCA1* were heavily methylated in A2780 and CP70 ovarian cancer cells, while such methylation was either undetectable or low in IOSE cells or other ovarian cancer cells (Figure 1C). Taken together, these results suggested that promoter hypermethylation may be responsible for the transcriptional repression of *ABCA1* in ovarian cancer.

### Depletion of *ABCA1* increases cholesterol level in ovarian cancer cells

**Figure 2:**
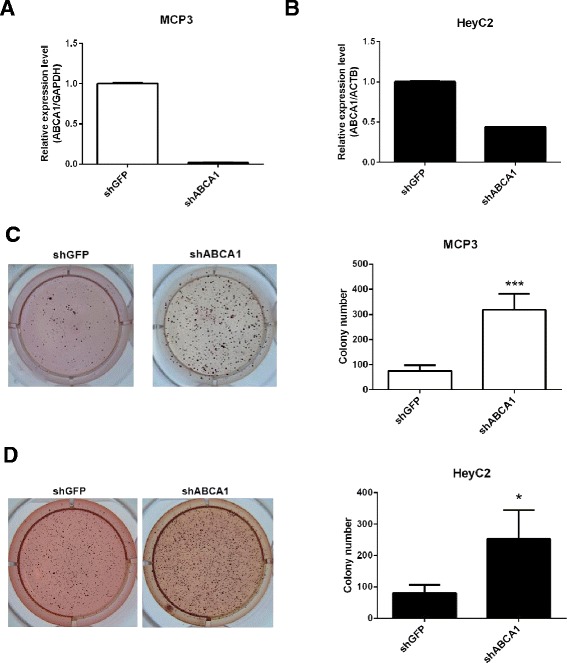
**Effects of** ***ABCA1*** **knockdown on cell growth.** Real-time RT-PCR expression of *ABCA1* in **(A)** MCP3 and **(B)** HeyC2 cells infected by lentivirus against shGFP (control) or shABCA1. Each bar represents mean ± SD. The growth of *ABCA1* knockdown **(C)** MCP3 and **(D)** HeyC2 cells was examined by soft agar assay. Quantitative analysis of the soft agar assay is also shown. ***P < 0.001; *P < 0.05.

**Figure 3:**
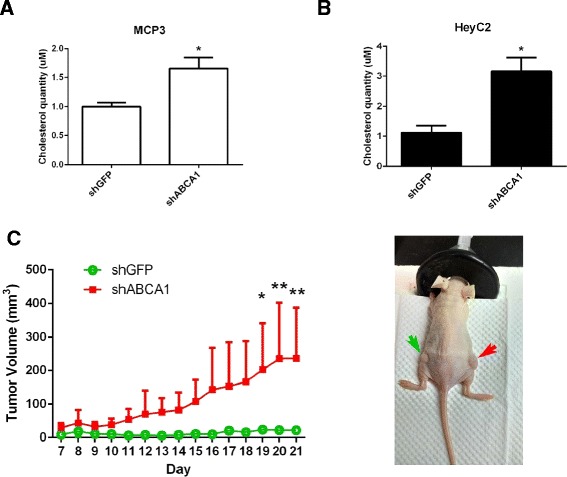
**Effects of** ***ABCA1*** **knockdown on cholesterol level and ovarian cancer growth** ***in vivo*** **.** The cholesterol level of *ABCA1* knockdown **(A)** MCP3 and **(B)** HeyC2 cells was measured by cholesterol quantitation assay. **(C)** The effect of *ABCA1* knockdown on tumor growth *in vivo* was determined by the nude mice model. HeyC2 cells stably infected with shABCA1 (red arrow) or shGFP (green arrow) were injected subcutaneously into athymic nude mice. One week later, tumor volumes were measured daily. From day 19, the volume of tumors with ABCA1 knockdown was significantly reduced as compared to vector controls (*P < 0.05; **P < 0.005). Data were expressed as mean ± SD (*n* = 3).

As *ABCA1* is responsible for cholesterol efflux, we first measured the effect of *ABCA1* depletion on the cholesterol level of ovarian cancer cells. Lentiviral knockdown of *ABCA1* was performed in MCP3 (Figure 2A) and HeyC2 (Figure 2B) ovarian cancer cells. The result showed that more than 50% repression of *ABCA1* was observed in these cells. As expected, depletion of *ABCA1* resulted in a significant accumulation of cholesterol in MCP3 (Figure 3A, *P* < 0.05) and HeyC2 (Figure 3B, *P* < 0.05) cells.

### Depletion of *ABCA1* promotes cell growth in ovarian cancer cells *in vitro* and *in vivo*

Dysregulation of cholesterol homeostasis has been shown to be related to cancer. Therefore, we hypothesized that *ABCA1* might act as a tumor suppressor in ovarian cancer. To confirm this hypothesis, we examined the effect of *ABCA1* depletion on the growth of ovarian cancer cells. Interestingly, depletion of *ABCA1* resulted in an increased cell growth in MCP3 (Figure 2C, *P* < 0.001) and HeyC2 (Figure 2D, *P* < 0.05) cells as demonstrated by colony formation assay. Further *in vivo* experiments also demonstrated that significant increased tumor growth was observed in ABCA1-depleted HeyC2 cells as compared to control (Figure 3C).

### Hypermethylation of *ABCA1* associates with tumor progression in ovarian cancer patients

**Figure 4:**
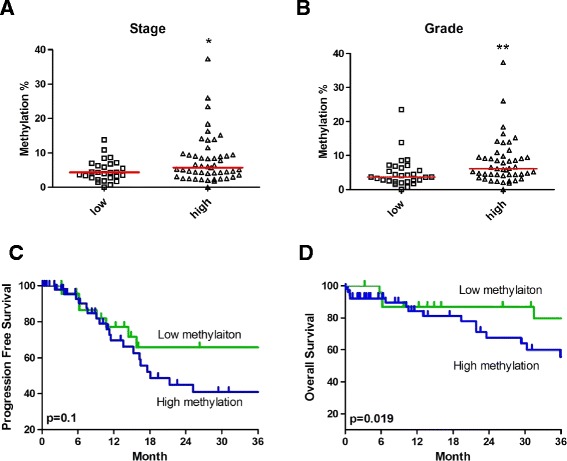
**Association between ABCA1 methylation and tumor progression.** Dot plot showing the association between *ABCA1* methylation in different **(A)** stages and **(B)** grades in 76 ovarian cancer patient samples. Methylation of *ABCA1* was determined by bisulfite pyro-sequencing. Low stage and low grade represented FIGO I and II and grade 1–2, respectively. While high stage and high grade represented FIGO III and IV and grade 3, respectively. *P < 0.05 by the Mann-Whitney *U* test. Kaplan-Meier analysis of *ABCA1* methylation for **(C)** progression-free survival and **(D)** overall survival in 76 ovarian cancer patient samples is shown. Patients were grouped according to methylation of *ABCA1* of 3%, which is based on the methylation level of IOSE cells. Patients with “high” *ABCA1* methylation (>3% methylation) have significant shorter overall survival (*P* = 0.019) but not progression-free survival than patients with “low” *ABCA1* methylation. Log-rank *P* values are shown.

Having demonstrated that promoter hypermethylation of *ABCA1* can be observed in ovarian cancer cell lines, we then proceeded to examine if such epigenetic event can also be observed in ovarian cancer patient samples (Table 1, Figure 4A, B). Results from bisulphite pyro-sequencing showed that patients with a higher stage (*P* = 0.026) and grade (*P* = 0.013) but not age have a significantly higher level of *ABCA1* methylation.

**Table 1:** **Association between methylation of** ***ABCA1*** **and clinicopathological features of 76 ovarian cancer patients**

	**Methylation %**	***P***
Age		
≧60	5.2 ± 3.01^a^ (53/76)^b^	0.379
<60	6.71 ± 5.34 (23/76)	
Stage^c^		
Low	4.36 ± 2.10 (27/76)	
High	6.52 ± 4.44 (49/76)	*0.026* ^e^
Grade^d^		
Low	4.71 ± 3.62 (29/76)	
High	6.26 ± 3.77 (47/76)	*0.013*

^a^Mean ± SD.

^b^Number in parentheses represents the number of cases.

^c^Low and high stages are defined as FIGO I and II and FIGO III and IV, respectively.

^d^Low and high grades are defined as grade 1–2 and grade 3, respectively.

^e^Italicized value indicates *P* < 0.05.

### Hypermethylation of *ABCA1* associates with poor prognosis in ovarian cancer patients

To further investigate if *ABCA1* methylation was predictive of survival in ovarian cancer patients, we performed Kaplan-Meier survival analyses. Using the baseline methylation level of *ABCA1* in IOSE cells (3%) as a cutoff, a significant association between patients with high *ABCA1* methylation and shorter overall survival (OS; *P* = 0.019) but not progression-free survival (PFS; *P* = 0.1) was observed (Figure 4C, D).

Because the above approaches based on a cutoff value may have biased the data analysis, the Cox proportional hazards model was used to analyze the predictive value of *ABCA1* methylation and other clinicopathological parameters on OS and PFS (Table 2). As expected, a higher stage (OS: hazard ratio: 11.321, *P* = 0.02; PFS: hazard ratio: 14.278, *P* < 0.01) and grade (OS: hazard ratio: 22.559, *P* = 0.03; PFS: hazard ratio: 20.586, *P* < 0.01) but not age was significantly associated with poor prognosis. Interestingly, methylation of *ABCA1* is also associated with poor patient outcome in ovarian cancer (OS: hazard ratio: 1.106, *P* = 0.033; PFS: hazard ratio: 1.096, *P* = 0.013). However, this association was not observed in multivariate analysis, thus suggesting that other factors are also involved in the survival of ovarian cancer patients (Table 3). Taken together, methylation of *ABCA1* is partially associated with poor prognosis in ovarian cancer patients.

**Table 2:** **Univariable analysis of survival by the Cox proportional hazards model**

**Variable**	**Overall survival**		**Progression-free survival**	
	**HR (95% CI)**	***P***	**HR (95% CI)**	***P***
*ABCA1* methylation	1.106 (1.008–1.213)	*0.033* ^a^	1.096 (1.020–1.179)	*0.013*
Age	2.014 (0.712–5.701)	0.187	1.309 (0.579–2.963)	0.518
Stage	11.321 (2.48–51.679)	*0.02*	14.278 (4.233–48.154)	<*0.01*
Grade	22.559 (2.849–178.624)	*0.03*	20.586 (4.817–87.977)	<*0.01*

^a^Italicized value indicates *P* < 0.05.

**Table 3:** **Multivariate analysis of survival by the Cox proportional hazards model**

	**PFS**		**OS**	
	**HR (95% CI)**	***P***	**HR (95% CI)**	***P***
ABCA1 methylation	1.013 (0.960–1.069)	0.636	1.016 (0.965–1.069)	0.554
Age	0.993 (0.961–1.025)	0.665	1.019 (0.988–1.051)	0.233
Stage	1.633 (1.159–2.302)	0.005	2.330 (1.196–4.538)	0.013
Grade	1.578 (0.557–4.473)	0.390	1.538 (0.340–6.963)	0.577

### Low expression of ABCA1 associates with poor prognosis in ovarian cancer patients

**Figure 5:**
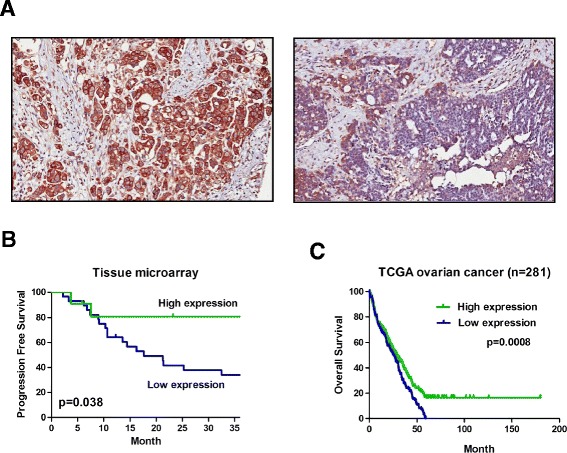
**Association between expression of** ***ABCA1*** **and survival in ovarian cancer patients.** Expression of ABCA1 in 55 ovarian cancer patient samples was determined by IHC in tissue microarray. **(A)** Representative image of ovarian cancer showing high (left panel) and low (right panel) ABCA1 expression on the cell membrane or cytoplasm (×400). **(B)** Kaplan-Meier analysis found that patients with low ABCA1 expression have shorter progression-free survival than patients with high ABCA1 expression (*P* = 0.038). **(C)** Similar results can be observed in TCGA ovarian cancer RNA-Seq dataset that patients with low expression of ABCA1 are associated with shorter overall survival (*P* = 0.0008). Log-rank *P* values are shown.

To further investigate the association between expression of ABCA1 and survival of ovarian cancer patients, we performed tissue microarray on a different cohort containing 55 ovarian patient samples (Table 4, Figure 5A). Although expression of ABCA1 is not associated with any clinicopathological parameters of the samples (Table 5), patients with low expression of ABCA1 are associated with shorter progression-free survival (Figure 5B). Similarly, analysis of TCGA ovarian cancer RNA-Seq dataset [14] also found that patients with lower expression of ABCA1 are associated with shorter overall survival (Figure 5C).

**Table 4:** **Summary of clinicopathological data of 55 ovarian cancer patients in tissue microarray**

	**Ovarian cancer (** ***n*** **= 55)**
Age at diagnosis (years)	
Median	51
Range	18–81
Stage	
FIGO (I–II)	22
FIGO (III–VI)	33
Grade	
I	9
II	14
III	32
Pathological type	
Clear cell	4
Serous	37
Mucinous	11
Endometroid	3
Median survival (months)	
PFS	25.3
OS	36.7

*PFS* progression-free survival, *OS* overall survival.

**Table 5:** **Association between expression of** ***ABCA1*** **and clinicopathological features of 55 ovarian cancer patients**

	**Expression score**		
	**High expression**	**Low expression**	
Age			
≧60	11^a^ (20%)	26 (47.2%)	
<60	5 (9.1%)	13 (23.6%)	0.572
Stage			
Low	6 (10.9%)	16 (29.1%)	
High	10 (18.2%)	23 (41.8%)	0.528
Grade			
Low	3 (5.5%)	6 (10.9%)	
High	13 (23.6%)	33 (60%)	0.522

^a^Number of cases.

## Discussion

In the current study, we demonstrated promoter hypermethylation of *ABCA1*, a cholesterol transporter in ovarian cancer cell lines and patient samples. This is a cancer-specific event as methylation of *ABCA1* was not observed in IOSE and normal ovarian surface epithelial (NOSE) cells. Methylation of *ABCA1* was also negatively associated with its expression and increased cell growth *in vitro* and *in vivo*. Importantly, ovarian cancer patients with higher *ABCA1* methylation were associated with shorter survival. Result from tissue microarray and TCGA ovarian cancer RNA-Seq dataset also demonstrated that ovarian cancer patients with lower ABCA1 expression were associated with shorter survival. Our results were also confirmed by a recent finding that *ABCA1* is epigenetically silenced by promoter methylation in prostate cancer cells. Hypermethylation of *ABCA1* is associated with high-grade prostate cancer [15]. Taken together, these results suggest that *ABCA1* may be a tumor suppressor and are epigenetically silenced in human cancer.

ABCA1, which belongs to the ATP-binding cassette (ABC) protein family, is responsible for cholesterol efflux and metabolism. It has been shown to be essential for the synthesis of high-density lipoprotein (HDL) particles by exporting cellular cholesterol out of the cell. Recent studies suggested that ABCA1 may be a tumor suppressor [16]. For example, Smith and Land demonstrated that overexpression of ABCA1 in colon cancer cells resulted in a decrease of cellular cholesterol and inhibition of tumor growth *in vitro* and *in vivo*. This growth inhibition may be due to apoptosis, as overexpression of ABCA1 enhanced cytochrome C release from mitochondria.

Expression of *ABCA1* has been previously shown to be activated by the nuclear receptor liver X receptor (LXR) [17]. Interestingly, TGF-β could significantly increase the expression of *ABCA1* through activation of LXR signaling [18]. Together with our previous finding that *ABCA1* is a TGF-β target in IOSE cells, it is thus suggested that the growth inhibitory effect of TGF-β may be partly due to overexpression of *ABCA1* in IOSE cells. Epigenetic alteration of *ABCA1* may contribute to the resistance of TGF-β-mediated growth suppression in ovarian cancer cells [9].

Dysregulation of cholesterol homeostasis has been known to be related to cancer [19]. Several studies have demonstrated an elevated level of cholesterol in tumor as compared to normal tissue [20,21]. Recently, Li et al. also demonstrated that ovarian cancer patients with higher serum low-density lipoprotein (LDL) level were significantly associated with shorter progression-free survival and overall survival [22]. It may be due to the fact that lipid serves as an energy source for promoting cell growth and metastasis in ovarian cancer [23]. In this regard, numerous studies have demonstrated that cholesterol-lowering agents, such as statins, exhibit anti-tumor activity against various cancers [24]. For example, statins have been shown to reduce tumor growth in xenograft models [25,26]. In ovarian cancer, a clinical study demonstrated that patients with statins are associated with improved survival [27]. This may be due to the growth inhibition and augmentation of apoptosis by statins in ovarian cancer cells [28-30]. Furthermore, inhibition of HMG-CoA reductase by statins can also lead to a decreased level of mevalonate, which is important for the synthesis of isoprenoids including geranyl pyrophosphate (GPP) and farnesyl pyrophosphate (FPP) [24,31]. Proper activation of several signaling molecules such as small GTP-binding proteins Ras and Rho require prenylation of GPP and FPP. Thus, blocking of cholesterol metabolism would reduce cancer growth by suppression of the proliferation signal.

## Conclusions

Promoter methylation of *ABCA1* was observed in ovarian cancer cell lines and patient samples. Ovarian cancer patients with higher methylation and lower expression of *ABCA1* were associated with shorter survival. The tumor suppressor function of *ABCA1* in ovarian cancer warrants further investigation.

## Methods

### Patient samples

Seventy-six treatment-naive ovarian cancer samples were obtained from Tri-Service General Hospital, Taipei, Taiwan (Table 6). Eight NOSE cell samples were acquired from patients during surgery for benign gynecological disease at either the Tri-Service General Hospital or Indiana University, USA. For this cohort of ovarian cancer patient samples, the median age at the time of diagnosis was 54 years (range, 18–90 years). Forty-nine cases (64.5%) were high stage (International Federation of Gynecology and Obstetrics (FIGO) stages III and IV), and 27 cases (35.5%) were low stage (FIGO stages I and II). Forty-seven cases (61.8%) were grade III, 13 cases (17.1%) were grade I, and 16 cases (21.1%) were grade II. All of the studies involving human ovarian epithelial samples were approved by the institutional review boards of the Tri-Service General Hospital, Taiwan, and Indiana University.

**Table 6:** **Summary of clinicopathological data of 76 ovarian cancer patients**

	**Ovarian cancer (** ***n*** **= 76)**
Age at diagnosis (years)	
Median	54
Range	18–90
Stage	
FIGO (I–II)	27
FIGO (III–VI)	49
Grade	
I	13
II	16
III	47
Pathological type	
Serous	48
Mucinous	23
Endometroid	5
Median survival (months)	
PFS	11.51
OS	17.42

*PFS* progression-free survival, *OS* overall survival.

### Cell culture

IOSE cells were derived by transducing the catalytic subunit of human telomerase and the papilloma virus subunit E7 into primary ovarian epithelial cells, as described previously [32]. The cells were maintained in a 1:1 mixture of Medium 199 (Sigma, St. Louis, MO) and 105 (Sigma) supplemented with 10% fetal bovine serum (FBS) (Invitrogen, Carlsbad, CA), 400 ng/ml hydrocortisone (Sigma), 10 ng/ml EGF, and 50 units/ml of penicillin/streptomycin (Invitrogen). The ovarian cancer cell lines A2780, CP70, MCP2, and MCP3 were propagated in RPMI 1640 (Invitrogen) containing 10% FBS. HeyC2 cells were cultured in Dulbecco’s modified Eagle’s medium (DMEM) containing 5% FBS, 1% NEAA, 1% Gln, and 1% HEPES. SKOV3 cells were cultured in McCoy’s 5A containing 10% FBS, 1% NEAA, 1% Gln, and 1% HEPES.

### DNA extraction, RNA extraction, and quantitative reverse transcription-PCR

DNA was extracted with the Tissue & Cell Genomic DNA Purification Kit (GeneMark, Taiwan). It was eluted in 50 μl distilled water and stored at −20°C until use. Total RNA from cell lines was extracted using TRIzol (Invitrogen), as previously described. Briefly, 1 μg of total RNA was treated with DNase I (amplification grade, Invitrogen) before first-strand cDNA synthesis using reverse transcriptase (Superscript II RT, Invitrogen). The real-time PCR reactions were carried out using the ABI StepOne real-time PCR system (Applied Biosystems, Foster City, CA) with specific primers (Additional file 1: Table S1). The relative expression of *ABCA1* was calculated using the comparative Ct method.

### Bisulfite conversion and pyro-sequencing

Bisulphite pyro-sequencing was performed as described previously [11]. Briefly, 0.5 μg of genomic DNA was bisulfite-modified using the EZ DNA Methylation Kit (Zymo Research, Orange, CA), according to the manufacturer’s protocol. The bisulfite-modified DNA was subjected to PCR amplification using a tailed reverse primer in combination with a biotin-labeled universal primer. The PCR and sequencing primers were designed using PyroMark Assay Design 2.0 (Qiagen GmbH, Hilden, Germany). The *ABCA1* TSS (−90 to +190) was PCR-amplified with specific primers (Additional file 1: Table S1) in a 25-μl reaction containing 2× RBC SensiZyme Hotstart Taq premix (RBC Bioscience, Taiwan). Prior to pyro-sequencing, 1.5 μl of each PCR reaction was analyzed on 1% agarose gel. The pyro-sequencing was performed on the PyroMark Q24 instrument (Qiagen) using the Pyro Gold Reagents (Qiagen), according to the manufacturer’s protocol. The methylation level of 11 CpG sites, which are located −13 to +95 with respect to the TSS, was measured. The methylation percentage of each cytosine was determined using the fluorescence intensity of cytosines divided by the sum of the fluorescence intensity of cytosines and thymines at each CpG site. *In vitro* methylated DNA (Millipore) was included as positive control for pyro-sequencing.

### Knockdown of ABCA1 by shRNA

The small hairpin RNA (shRNA) of ABCA1 were acquired from the National RNAi Core Facility Platform at the Institute of Molecular Biology/Genomic Research Center, Academia Sinica, Taiwan. Briefly, 293T cells were transfected with shRNA (TRCN0000029093), pMDG, and pCMVR 8.91 using the ProFection Mammalian Transfection System (Promega) to prepare the shABCA1 lentivirus. Infected ovarian cancer cells were selected by incubating with puromycin (2 μg/ml; Sigma) for at least 2 days.

### Soft agar assay for colony formation

Trypsinized cells (1,000 cells) were seeded and mixed in 1.5 ml of 0.35% top layer agar supplemented with DMEM with 10% FBS. This suspension was overlaid on the bottom layer of 0.5% agar in DMEM with 10% FBS in a six-well plate. Plates were allowed to solidify and then incubated at 37°C for around 3 weeks. Colony formation was monitored daily by microscopic observation. At the end of the experiments, the plates were stained with iodonitrotetrazolium (INT) stain (Sigma) for 48 h at 37°C. The number of colonies was counted.

### Immunohistochemical analysis on ovarian tissue microarray

The paraffin-embedded ovarian cancer patients’ tissue microarray were prepared and retrieved from the Department of Pathology, Tri-Service General Hospital as described previously [33]. The tissue microarray contained 55 samples of ovarian cancer patients (Table 4). The immunohistochemistry procedure followed a standard protocol, using a rabbit polyclonal anti-human ABCA1 antibody (NB400-105, Novus Biologicals). All tissue microarray slides were examined and scored by two pathologists. Immunohistochemistry (IHC) scoring was determined by distribution of positively stained cell × intensity of the staining. A scoring of 60 was used as a cutoff value.

### *In vivo* tumorigenicity assay

A total of three, 8-week-old, athymic nude mice (BALB/cByJNarl) were obtained from the National Laboratory Animal Center, Taiwan. All mice were kept under specific pathogen-free conditions using a laminar airflow rack with free access to sterilized food and autoclaved water. All experiments were performed under license from Animal Experimentation Ethics Committee of the National Chung Cheng University. HeyC2 cells (1 × 10^6^) stably infected with pLKO.1/shABCA1 or pLKO.1/shGFP were re-suspended in 0.1 ml of medium and Matrigel (BD Biosciences, San Jose, CA) mixture (1:1). The cell suspension was then injected subcutaneously into the flank of each mouse (day 0). Tumor size was measured daily with calipers in length (*L*) and width (*W*). Tumor volume was calculated using the formula (*L* × *W*^2^/2). At the end of the experiment, all mice were sacrificed by cervical dislocation.

### Analysis of cellular cholesterol content

Ovarian cancer cells (1 × 10^4^) infected with shABCA1 or shGFP were seeded in a six-well dish and cultured for 48 h. After 48 h, the cells were washed with cold PBS twice and scraped. The cells were re-suspended in 10% RIPA buffer (50 mM HEPES-KOH, pKa 7.55, 500 mM LiCl, 1 mM EDTA, 0.1% NP-40, and 0.07% Na-deoxycholate) and kept on ice for 15 min. Cholesterol content was determined by the Amplite™ Fluorimetric Cholesterol Quantitation Kit (AAT Bioquest, Sunnyvale, CA) according to the manufacturer’s protocol. Fluorescence of cholesterol was detected by Ex/Em at 540/590 nm.

### Statistical analysis

PFS and OS were assessed by Kaplan-Meier analysis using the log-rank test. Progression-free survival was defined as the duration from the day of diagnosis or chemotherapy to the detection of new lesions or progression of residual lesions. Overall survival was defined as the duration from the day of diagnosis to death. A DNA methylation level at 3%, which is the level of methylation in OSE cells, was used as a cutoff. Fisher’s exact test or the Mann-Whitney *U* test was also used to compare parameters of different groups. All statistical calculations were performed using the statistical package SPSS version 13.0 for Windows (SPSS, Inc., Chicago, IL). *P* < 0.05 was considered to be statistically significant. For TCGA ovarian cancer RNA-Seq dataset, a mean RPKM value of 4 was used as a cutoff.

## Additional file

Additional file 1: Table S1. Primer sequences used in the study. **Table S1.** lists all the primers for RT-PCR and bisulphite pyro-sequencing.
