# Supplementary material for: Hypermethylation of the TGF-β target, ABCA1 is associated with poor prognosis in ovarian cancer patients
Source: Clin Epigenetics. 2015 Jan 14;7(1):1. doi: 10.1186/s13148-014-0036-2 (PMC4307187; doi:10.1186/s13148-014-0036-2)
Supplement: Additional file 1: Table S1. — Primer sequences used in the study. Table S1. lists all the primers for RT-PCR and bisulphite pyro-sequencing. [file 13148_2014_36_MOESM1_ESM.doc]

Additional file 1: Table S1 Primer sequences used in the study

|  | Sequence (5’ to 3’) |
| --- | --- |
| **RT primer** |  |
| ABCA1 forward | TCTCACCACTTCGGTCTCCATG |
| ABCA1 reverse | CCTCGCCAAACCAGTAGGACTT |
| GAPDH forward | CCCCTTCATTGACCTCAACTACAT |
| GAPDH reverse | TCACCATCTTCCAGGAGCG |
| **Pyro-sequencing primer** | |
| ABCA1 forward | GGAGGAGGGAGAGTATAGGT |
| ABCA1 reverse | ggtcgtcagactgtcgatgaagccCTCCTACCCCTTAACAAACCTTC |
| Biotinated UNIVR^*^ | ggtcgtcagactgtcgatgaagcc |
| Sequencing primer | GAATTTATAAAAGGAATTAGTT |

* Primer sequence of the 5’tailed universal primer (UNIVR) is shown as lower case.
